# Synthesis and Characterization of Temperature- and *pH*-Responsive PIA-b-PNIPAM@Fe_3_O_4_ Nanocomposites

**DOI:** 10.3390/nano15131041

**Published:** 2025-07-04

**Authors:** Swati Kumari, Cayla Cook, Fatema Tarannum, Erick S. Vasquez-Guardado, Olufemi Ogunjimi, Keisha B. Walters

**Affiliations:** 1 Operations Food Safety, Amazon, Santa Clara, CA 95054, USA; swati.kum05@gmail.com; 2 Hazen and Sawyer, Tempe, AZ 85282, USA; ccook@hazenandsawyer.com; 3 Ralph E. Martin Department of Chemical Engineering, University of Arkansas, Fayetteville, AR 72701, USA; tarannum@uark.edu (F.T.); ogunjimi@uark.edu (O.O.); 4 Department of Chemical and Materials Engineering, University of Dayton, Dayton, OH 45469, USA; evasquez1@udayton.edu

**Keywords:** stimuli responsive, poly(itaconic acid), poly(N-isopropyl acrylamide), nanocomposite, temperature, *pH*

## Abstract

Stimuli-responsive polymers (SRPs) have garnered significant attention in recent decades due to their immense potential in biomedical and environmental applications. When these SRPs are grafted onto magnetic nanoparticles, they form multifunctional nanocomposites capable of various complex applications, such as targeted drug delivery, advanced separations, and magnetic resonance imaging. In this study, we employed a one-step hydrothermal method using 3-aminopropyltrimethoxysilane (APTES) to synthesize APTES-modified Fe_3_O_4_ nanoparticles (APTES@Fe_3_O_4_) featuring reactive terminal amine groups. Subsequently, via two consecutive surface-initiated atom transfer radical polymerizations (SI-ATRP), *pH*- and temperature-responsive polymer blocks were grown from the Fe_3_O_4_ surface, resulting in the formation of poly(itaconic acid)-block-poly(N-isopropyl acrylamide) (PIA-b-PNIPAM)-grafted nanomagnetic particles (PIA-b-PNIPAM@Fe_3_O_4_). To confirm the chemical composition and assess how the particle morphology and size distribution of these SRP-based nanocomposites change in response to ambient *pH* and temperature stimuli, various characterization techniques were employed, including transmission electron microscopy, differential light scattering, and Fourier transform infrared spectroscopy. The results indicated successful synthesis, with PIA-b-PNIPAM@Fe_3_O_4_ demonstrating sensitivity to both temperature and *pH*.

## 1. Introduction

Polymers are widely utilized as materials due to their exceptional properties, including high strength, durability, flexibility, and ease of processing, which make them suitable for various applications, including consumer products, coatings, electronics, sensors, and biomedical devices [1,2,3,4,5,6,7]. To function as smart materials, however, these polymers must generally demonstrate reversible and measurable responses to one or more external stimuli such as mechanical stress, heat, light, gases, electricity, and *pH* changes. Stimuli-responsive polymers (SRPs), sometimes referred to as “smart” polymers, incorporate functional groups or moieties that enable controlled, reversible changes in their physical or chemical properties in response to these stimuli [8]. For instance, thermoresponsive polymers like poly(N,N-diethylacrylamide) (PDEAAm) shift from being hydrophilic to hydrophobic with temperature changes [9], while *pH*-sensitive polymers like poly(acrylic acid) (PAA) display a change in their solubility as a function of *pH* [10,11]. These responsive behaviors have significantly expanded the potential of these polymers for application in fields like medicine, biosensing, drug delivery and diagnostics, image targeting, tunable catalysis, self-healing materials, and colloids, offering controlled release, enhanced bioavailability, improved therapeutic specificity, reduced toxicity, and integrated theranostic capabilities [9,12,13,14,15,16,17,18,19].

A wide variety of water-soluble, and biocompatible polymers—such as poly(ethylene glycol) (PEG) [20,21], poly(ethylene oxide) (PEO) [22], dextran [23,24], poly(acrylic acid) (PAA) [25,26], poly(methacrylic acid) [27,28], and poly(L-histidine) [29]—have been used in the fabrication of SRPs due to their tunable physicochemical properties. Among them, poly(N-isopropylacrylamide) (PNIPAM) is a well-known SRP due to its temperature responsiveness [30,31,32], characterized by hydrophilic amide (-CONH-) groups and hydrophobic isopropyl (-CH(CH_3_)_2_) side chains. With a lower critical solution temperature (LCST) of ~32 °C [33]—just below human body temperature (~37 °C)—PNIPAM undergoes a hydrophilic-to-hydrophobic transition. Below its LCST, PNIPAM exists in a hydrated, swollen state capable of hydrogen bonding with hydrophilic drugs [33], whereas above its LCST, hydrophobic interactions dominate, causing the polymer chains to collapse and release their payload [34]. Copolymerization with other monomers can further tailor both its LCST and *pH* responsiveness to specific applications [35]. Complementarily, poly(itaconic acid) (PIA) can contribute significant *pH* sensitivity due to its ionizable carboxylic acid groups (*pKa* ≈ 4.3 and 5.6). Despite its poor solubility in most organic solvents, PIA’s hydrophilicity and biocompatibility [36,37] make it a promising candidate for sustainable and stimuli-responsive systems. In this study, the combination of PIA and PNIPAM results in a dual-stimuli-responsive nanocomposite, suitable for targeted drug delivery, diagnostics, and environmental applications.

The synthesis of SRPs, particularly PNIPAM, has been achieved through various polymerization techniques, such as free radical polymerization (FRP), ionic polymerization, redox polymerization, radiation polymerization, and atom transfer radical polymerization (ATRP) [38]. An extension of ATRP, known as surface-initiated ATRP (SI-ATRP), facilitates spatially controlled polymer growth, high grafting densities, and uniform brush morphologies, making it particularly well-suited for fabricating surface-confined stimuli-responsive systems [39]. The integration of SRPs with nanomaterials, like carbon nanotubes (CNTs) [40,41,42], graphene [43] and especially SPIONPs, for their high surface area and superparamagnetism, has led to the development of multifunctional platforms for targeted drug delivery, diagnostics, bioimaging, magnetic resonance imaging (MRI), and magnetically controlled therapies [44,45,46,47,48,48,49,50,51]. For instance, PNIPAM-coated SPIONPs synthesized via SI-ATRP enabled ~90% doxorubicin (DOX) release at *pH* 5 and 42 °C while remaining stable under physiological conditions, demonstrating potential for MRI-guided cancer therapy [52]. A hybrid of PNIPAM–poly(ethylene glycol)methacrylate (PEGMA) microgels grafted onto MIL-101(Cr) MOFs showed improved thermoresponsive drug delivery for osteoarthritis treatment by leveraging tunable LCST behavior [53]. Similarly, poly(3-sulfopropyl methacrylate potassium salt) (PSPMK) brushes grafted onto UiO-66-NH_2_ MOFs loaded with aspirin enabled sustained anti-inflammatory drug release for osteoarthritis treatment [54]. Ramanujan et al. developed a PNIPAM–MBAm (N,N′-methylenebis(acrylamide)) hydrogel embedded with SPIONPs for alternating magnetic field (AMF)-triggered doxorubicin release through localized heating-induced contraction to enable controlled drug delivery [55]. In another study, Toma et al. designed a PNIPAM-based surface plasmon resonance (SPR) biosensor with an indium tin oxide (ITO) microheater that modulated signals via temperature-driven volume changes [33]. A chitosan/reduced graphene oxide/SPIONP nanocomposite developed by Karthika et al. demonstrated high drug-loading capacity with magnetically controlled release [56]. Table 1 provides a comprehensive overview of some recent studies on stimuli-responsive polymer composites, highlighting their synthesis strategies, their functionalization techniques, and their tunable application because of external stimuli such as *pH*, temperature, and magnetic fields. In this present study, a two-step SI-ATRP strategy was applied to synthesize block copolymers on superparamagnetic iron oxide nanoparticles (SPIONPs), aiming to improve the performance of PNIPAM-based SRPs.

Despite their potential, SPIONPs’ intrinsic tendency to agglomerate, driven by strong dipole–dipole interactions, poses challenges for colloidal stability. Therefore, surface modification is crucial to enhance their dispersibility and biocompatibility and ensure uniform distribution within polymeric composite networks. Aminopropyltrimethoxysilane (APTES), a widely used silane coupling agent, contains three hydrolyzable ethoxy groups that support silanization, with stable Si–O bonds forming with the Fe_3_O_4_ nanoparticle surface and the terminal amine group (–NH_2_) acting as a reactive site for the subsequent grafting of polymers [45] such as PIA and PNIPAM. This functionalization enhances the integration and dispersion of SPIONPs, thereby providing a stable platform for their direct use or further surface modification, such as the addition of a polymer brush.

In this study, initially, Fe_3_O_4_ nanoparticles were synthesized in-house using a one-step hydrothermal technique, followed by surface modification with 3-aminopropyltrimethoxysilane (APTES) to introduce reactive amine groups on the nanoparticle surface. Unlike previous studies that utilized commercially available nanoparticles, the in-house synthesis helped to minimize the breakage and aggregation of NPs. Then, two successive surface-initiated atom transfer radical polymerization (SI-ATRP) steps were performed to prepare a dual-responsive (*pH* and temperature) block copolymer, poly(itaconic acid)-block-poly(N-isopropylacrylamide) (PIA-b-PNIPAM), on the surface of APTES-modified Fe_3_O_4_ (APTES@Fe_3_O_4_) or amine-functionalized Fe_3_O_4_ NPs. APTES modification allows the polymer chains to extend outward from the nanoparticle surface, minimizing the entanglement and improving colloidal stability. The chemical composition, structure, *pH* and temperature responsiveness, and morphology of the PIA–b-PNIPAM@Fe_3_O_4_ nanoparticles were characterized using Fourier transform infrared spectroscopy (FTIR), dynamic light scattering (DLS), and transmission electron microscopy (TEM). Building upon our previous work involving PNIPAM-b-PIA grafted onto APTES@Fe_3_O_4_ via SI-ATRP [57] for thermoresponsive applications, this study further investigates the dual-stimuli-responsiveness (*pH* and temperature dependence) of these nanocomposites for potential biomedical applications. Specifically, the PNIPAM block can undergo a phase transition at physiological temperatures (~32 °C), enabling drug release in response to body heat, while the PIA block can respond to acidic environments, such as those found in tumor tissues or intracellular compartments (e.g., endosomes/lysosomes). This dual-trigger mechanism enhances site-specific release, minimizes premature drug leakage, improves drug bioavailability, and reduces systemic side effects. Therefore, the synthesized PIA-b-PNIPAM@Fe_3_O_4_ nanocomposites show strong potential for use in advanced drug delivery systems, including targeted drug delivery and magnetic hyperthermia ferrofluids (MHFs) for cancer therapy.

**Table 1:** Recent literature review on stimuli-responsive polymer composites with their intended applications.

Synthesis Method	Polymer Used	Functionalization	MNP	Nanocomposite	Intended Application	Reference
Graft polymerization	Chitosan and PEG	PNIPAM	-	Chitosan films	Medium suppuration wounds	Conzatti et al. [58]
One-step precipitation polymerization	PNIPAM	DNAzyme	-	PNIPAM/DNAzyme	Biocatalysts (bioassays and biosensors)	Li et al. [59]
Soap-free emulsion polymerization	PNIPAM	Thiol- and carboxyl-functionalization	Bimetallic (Cu/Pd)	PNIPAM-based bimetallic Cu/Pd	Hybrid catalytic materials in the synthesis of nitrogenous compounds with improved optical properties	Kakar et al. [60]
Thermal polymerization	Itaconic acid (IA)	Laponite RD	-	IA/Laponite RD hydrogels	Removal of cationic dyes	Huerta-Angeles et al. [61]
Radical polymerization	PNIPAM	PIA	Fe_3_O_4_-NPs	Folate conjugated Poly(NIPAM-co-IA)@Fe_3_O_4_	Nanocarrier for targeted doxorubicin delivery	Ghorbani et al. [62]
Hydrothermal approach	PNIPAM	-	Nickel ion doped iron oxide NPs	NiFe_2_O_4_-PNIPAM	Template for the efficient recovery of cefixime and methylene blue	Anushree et al. [63]
Free radical polymerization	Poly(NIPAM-co-AA)	APTES and Alanine	Ferrite and Magnetite MNPs	Poly(NIPAM-co-AA) immobilized on MNPs	Comparison between ‘grafting to’ and ‘in situ’ methods	Sakai et al. [64]
Copolymerization	PNIPAM	PIA	TiO_2_@SiO_2_	PNIPAM/PIA- TiO_2_@SiO_2_	Investigation in antibacterial activities against Gram-positive and Gram-negative bacteria (*S. aureus* and *E. coli*)	Mohamed and Hassabo [65]
Surface-initiated ATRP	PNIPAM	2-bromopropionamidepropyl tri- methoxysilane (BPTMS)	Fe_3_O_4_-NPs	SPION-PNIPAM	Contrast generation in MRI	Yar et al. [52]
Surface-initiated ATRP	PNIPAM	PIA	Fe_3_O_4_-NPs	Fe_3_O_4_@PIA-b-PNIPAM	Demonstration of thermo-responsive feature	Eyiler and Walters [57]

## 2. Experimental Section

### 2.1. Materials

Iron (III) chloride hexahydrate (FeCl_3_.6H_2_O, CAS # 10025-77-1, 97%), sodium hydroxide (NaOH, ≥98%, anhydrous pellets), N-isopropyl acrylamide (NIPAM, ≥99%), itaconic acid (IA, ≥99%), 2,2′-bipyridyl (bpy, ≥99%), anhydrous 2-propanol (≥99.5%), and 3-aminopropyltrimethoxysilane (APTES, CAS #13822-56-5, 97%) were procured from Sigma-Aldrich (St. Louis, MO, USA). Ammonium hydroxide (NH_4_OH), ethanol, trimethylamine (TMA, CAS #75-50-3, 40%), and 2-bromopropionyl bromide (BPB, CAS #563-76-8) were purchased from Thermo Fisher Scientific. All chemicals were used as received without further purification unless otherwise specified. Millipore (Burlington, MA, USA) (Type I) water with a resistivity of 18.2 MΩ·cm was used in all experiments.

### 2.2. Synthesis of APTES-Modified Fe_3_O_4_ Nanoparticles

**Figure 1:**
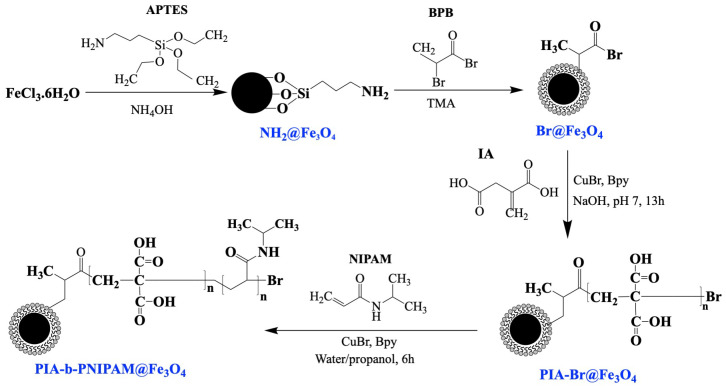
Schematic illustration of reaction mechanism for synthesizing APTES-modified Fe_3_O_4_ (APTES@Fe_3_O_4_) and PIA-b-PNIPAM@Fe_3_O_4_ via hydrothermal approach and surface-initiated ATRP (SI-ATRP). APTES: 3-aminopropyltrimethoxysilane; BPB: 2-bromopropionyl bromide; IA: itaconic acid; NIPAM: N-isopropyl acrylamide.

Superparamagnetic iron oxide nanoparticles (SPIONPs) were synthesized via a one-step hydrothermal approach incorporating APTES in order to achieve APTES-modified Fe_3_O_4_ nanoparticles bearing reactive terminal amine groups [45], as shown in Figure 1. A solution was prepared by dissolving 1.25 g of FeCl_3_·6H_2_O in 7.75 mL of deionized (DI) water, followed by stirring at 300 rpm for 10 min. Simultaneously, 6.25 mL of NH_4_OH was added dropwise under continuous stirring. The resulting mixture was then combined with 2.5 mL of APTES and transferred into a hydrothermal reactor, which was subsequently sealed. The reactor was placed in a preheated oven at 134 °C and maintained at this temperature for 3 h. After the reaction, the obtained product was washed three times with DI water and twice with ethanol using a 2T magnet for separation. The purified nanoparticles, denoted as APTES-modified Fe_3_O_4_ or APTES@Fe_3_O_4_, were then sonicated and dispersed in water at a final concentration of 1 mg.mL^−1^.

### 2.3. Surface-Initiated Block Copolymerization of Itaconic Acid (IA) and N-Isopropyl Acrylamide (NIPAM) on Fe_3_O_4_ Nanoparticles

An aliquot of 0.25 mL APTES@Fe_3_O_4_ or amine-functionalized Fe_3_O_4_ nanoparticles in aqueous solution was vacuum-dried in a Schlenk flask. TMA (83.6 µL, 0.5 mmol) obtained from a 40 wt.% aqueous solution was diluted to a total volume of 1 mL with water and added to the reaction mixture at approximately 0 °C. This was followed by the dropwise addition of BPB (52.4 µL, 0.5 mmol) dissolved in 5 mL of solvent. The reaction mixture was stirred overnight at room temperature. The bromine-initiated NPs (Br@Fe_3_O_4_) were then isolated using a 1.32 T magnet, washed with ethanol, sonicated three times, and subsequently dried under vacuum at room temperature.

Separately, a 1 M solution of IA (2.602 g, 20 mmol) was prepared in 15 mL of HPLC-grade water and deprotonated using NaOH (1.64 g) to adjust the *pH* to 7. Immediately after, bpy (62.5 mg, 0.4 mmol) and anhydrous 2-propanol (5 mL) were added, followed by sonication for 10 min. Dissolved oxygen was extracted using three freeze–pump–thaw cycles. Following the addition of Cu(I)Br (28.7 mg, 0.2 mmol) to the frozen solution, two additional cycles were performed. The solution was then left to polymerize for 13 h. The resulting PIA-grafted NPs (PIA-Br@Fe_3_O_4_) were purified via washing three times with water and left to settle under magnetic separation. The NPs were subsequently dried under vacuum.

Next, NIPAM (2.263 g, 20 mmol) was dissolved in a mixture of anhydrous 2-propanol/HPLC water (at 3:1 v/v, total volume at 20 mL). bpy (62.5 mg, 0.4 mmol), and Cu(I)Br (28.7 mg, 0.2 mmol) were then quickly added to the mixture after three freeze–pump–thaw cycles. Two additional freeze–pump–thaw cycles were performed to degas the mixture before 10 min sonication. The reaction mixture was polymerized for 6 h and then exposed to air. The resulting PIA-b-PNIPAM-grafted NPs (PIA-b-PNIPAM@Fe_3_O_4_) were separated using a magnet, and any remaining catalyst, unreacted monomer, and ungrafted polymers were removed through three successive washes with water and ethanol. Finally, the purified nanoparticles were dried under vacuum.

### 2.4. Characterization

The APTES-modified Fe_3_O_4_ and PIA-b-PNIPAM@Fe_3_O_4_ samples were characterized by attenuated total reflectance Fourier transform infrared (ATR-FTIR) spectroscopy using a Thermo Electron 6700 instrument equipped with a mercury–cadmium–telluride (MCT-A*) detector and a KBr beam splitter, controlled by OMNIC software (v8.1.10, Thermo Fisher Scientific Inc., Waltham, MA, USA). Spectra were collected over 800–4000 cm^−1^ at a resolution of 4 cm^−1^, averaging 256 scans using a MIRacle-ATR accessory (Pike Technologies (Fitchburg, WI, USA)) with a ZnSe/diamond crystal. The samples were deposited as solution droplets and allowed to dry on the crystal surface to form a thin film of nanoparticles.

A Zeta PALS analyzer (Brookhaven Instruments Corporation, BIC, Nashua, NH, USA) with a 659 nm laser was used to measure nanoparticle hydrodynamic size and distributions in water by dynamic light scattering (DLS) at a 90° detection angle. Before the measurements, samples were sonicated for 5 min and then allowed to stabilize in the cuvette for 3 min. Five consecutive 5-min runs were performed to determine particle size based on the effective diameter. Data collection and analysis were performed using BIC Particle Solutions software (v2.0).

Transmission electron microscopy (TEM) studies were carried out using a JEOL 100CXII operated at 100 kV, with high-resolution images obtained with a JEOL 2100 at 200 kV. For sample preparation, the nanoparticles were sonicated in an ultrasonic bath for 5 min; then, a droplet was deposited onto a carbon Formvar copper grid (Electron Microscopy Science, Hatfield, PA, USA) and air-dried in a ventilated hood.

## 3. Results and Discussion

**Figure 2:**
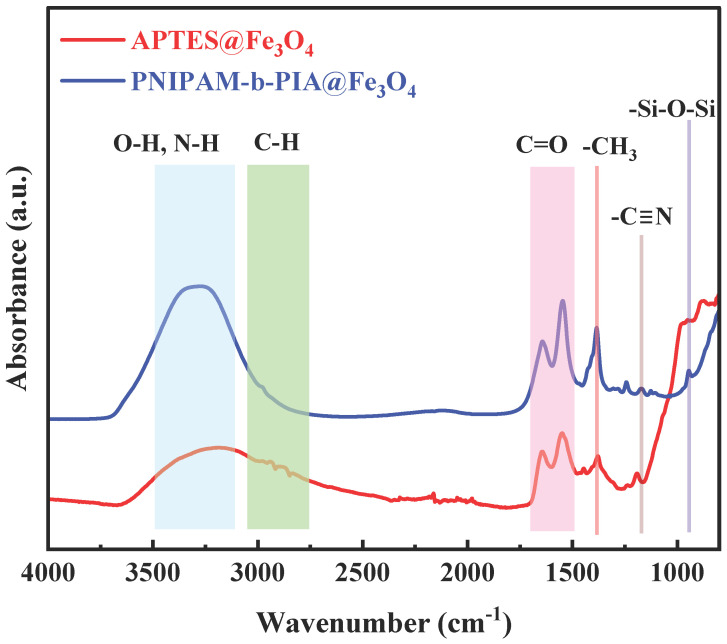
FTIR spectra of APTES@Fe_3_O_4_ (red spectrum) and PIA-b-PNIPAM@Fe_3_O_4_ (blue spectrum).

The successful functionalization of APTES-modified Fe_3_O_4_ (APTES@Fe_3_O_4_) and PIA-b-PNIPAM@Fe_3_O_4_ samples was confirmed through FTIR spectroscopy, as shown in Figure 2. The peaks in the 1100–1000 cm^−1^ range correspond to Si–O–Si stretching vibrations [66] from the APTES layer, verifying silanization or APTES attachment in the APTES-modified Fe_3_O_4_ sample. As APTES itself does not possess a carbonyl (C=O) group, the weak band near ~1600 cm^−1^ in the APTES@Fe_3_O_4_ spectrum arises from N–H bending of the surface –NH_2_ groups [67] or from adsorbed water. A broad –OH/N–H stretching band is observed around 3300–3400 cm^−1^, mostly from surface –NH_2_ and hydroxyl groups. Finally, after polymer grafting in the PIA-b-PNIPAM@Fe_3_O_4_ spectrum, the N–H stretching band becomes broader and more intense, shifting slightly to ~3280–3350 cm^−1^ [68], due to hydrogen bonding and increased amide content from PNIPAM and PIA chains. A clear and strong amide I band emerges at ~1650 cm^−1^, corresponding to C=O stretching, and an amide II band appears at ~1540 cm^−1^, corresponding to N–H bending and C–N stretching [68,69], which were absent in the APTES@Fe_3_O_4_. Moreover, a peak corresponding to the carboxylic acid C=O stretching in PIA, typically observed around 1710–1720 cm^−1^ [62,70], shifts to a lower wavenumber (~1695 cm^−1^) [71] upon hydrogen bonding with surface amines, further confirming polymer attachment. These spectral changes—especially the emergence of and shifts in amide and carboxyl bands—provide strong evidence for the successful surface grafting of PNIPAM-b-PIA onto Fe_3_O_4_ nanoparticles.

**Figure 3:**
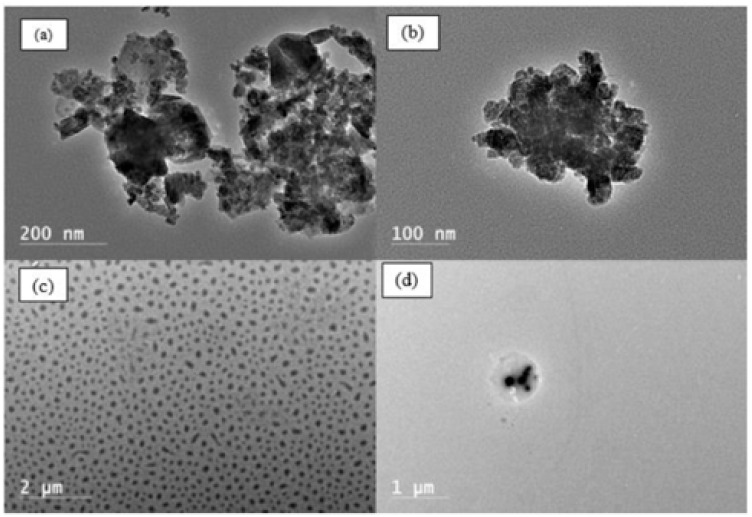
Transmission electron micrographs of APTES@Fe_3_O_4_ (**a**), PIA@Fe_3_O_4_ (**b**), and PIA-b-PNIPAM@Fe_3_O_4_ (**c**,**d**).

Transmission electron microscopy (TEM) was performed to investigate the morphological characteristics of both APTES-modified Fe_3_O_4_ nanoparticles and surface-functionalized SRPs following two successive surface-initiated atom transfer radical polymerization (SI-ATRP) reactions. Firstly, the polymerization of IA onto APTES@Fe_3_O_4_ was observed, as illustrated in Figure 3a,b. The TEM images reveal the presence of a diffuse, hazy layer surrounding the iron oxide cores, indicative of the polymeric shell formed by PIA (Figure 3b).

Subsequently, the synthesis of the block copolymer PIA-b-PNIPAM@Fe_3_O_4_ was confirmed by the polymerization of NIPAM, as shown in Figure 3c,d at different magnifications. The TEM micrographs distinctly depict the core–shell morphology, where the dark dense spherical core corresponds to Fe_3_O_4_ nanoparticles, and the surrounding lighter region corresponds to the PIA-b-PNIPAM structure. The observed particle size of ~1100 nm or ~1.1 µm confirms the significant increase in nanoparticle dimensions due to the successive polymerization steps, consistent with the hydrodynamic size measured by dynamic light scattering (DLS). The contrast variation between the core and shell regions in the TEM images further supports the successful encapsulation of the magnetic nanoparticles within the amphiphilic polymer matrix, reinforcing the efficiency of the SI-ATRP process in fabricating well-defined nanocomposites.

DLS data indicate that APTES@Fe_3_O_4_ nanoparticles had an average hydrodynamic diameter of 208 ± 2 nm. After grafting with PIA, the particle diameter increased significantly to 881 ± 8 nm, indicating the successful formation of PIA polymer shell around the magnetic core. Further modification to PIA-b-PNIPAM@Fe_3_O_4_ resulted in an additional size increase to 1164 ± 63 nm. This substantial growth suggests the significant incorporation of PNIPAM as the secondary polymer block. In addition, both PIA and PNIPAM polymers are expected to be hydrated due to their hydrophilic nature and ability to swell in an aqueous environment. The observation at the nanocomposite size aligns with expectations based on the polymer grafting process. Initially, the APTES coating on Fe_3_O_4_ provides a thin functional layer, minimally affecting the nanoparticle size. However, the introduction of a polyanionic polymer, PIA leads to a pronounced increase in hydrodynamic diameter due to electrostatic repulsion and hydration effects, which contribute to the overall expansion of the polymer corona. The subsequent grafting of PNIPAM further enlarges the nanoparticles, potentially due to additional hydration and chain extension in water at ambient temperatures.

Moreover, the DLS-measured hydrodynamic diameters correlate well with the TEM observations (as illustrated in Figure 3), where the core–shell structures of the nanoparticles are visually distinguishable. The difference between DLS and TEM measurements can be attributed to the hydration layer and polymeric swelling, as DLS assesses the hydrodynamic size in solution, while TEM provides a dry-state size measurement. This agreement between DLS and TEM confirms the successful stepwise surface modification of Fe_3_O_4_ nanoparticles with functional polymers, enhancing their applicability in biomedical and environmental applications.

**Figure 4:**
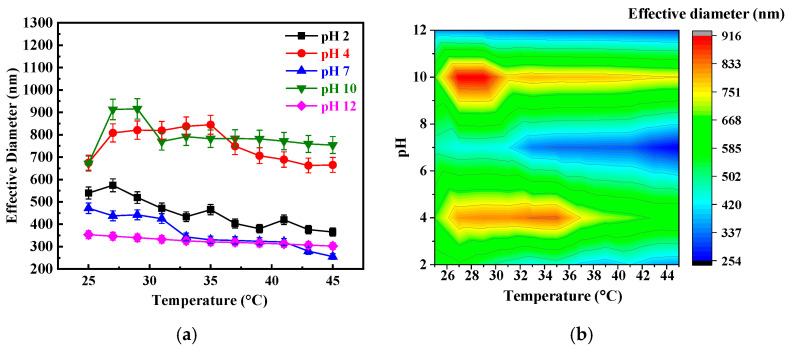
(**a**) Effective diameter as a function of temperature (25–45 °C) and *pH* (2–12) and (**b**) corresponding heat map of PIA-b-PNIPAM@Fe_3_O_4_ stimuli-responsive polymer composite measured from DLS. Color gradient corresponds to particle size (in nm), with red indicating maximum swelling and blue indicating minimum size due to the polymer chain collapse.

Furthermore, the thermo- and *pH*-responsive behavior of the PIA-b-PNIPAM@Fe_3_O_4_ composite was investigated using DLS across temperatures (25–45 °C) and *pH* values. Given PNIPAM’s LCST (~32 °C), changes in hydrodynamic diameter were monitored to track the phase transitions of the composite. Figure 4a illustrates how the effective hydrodynamic diameter of the PIA-b-PNIPAM@Fe_3_O_4_ changes with temperature and *pH*, offering valuable insights into the dual environmental responsiveness of the nanocomposite. A substantial 45% reduction in hydrodynamic diameter was observed as temperature increased at a neutral *pH* (*pH* 7), a phenomenon attributed to the thermoresponsive collapse of the PNIPAM shell above its LCST. Below 32 °C, PNIPAM adopts a hydrated, extended coil conformation due to strong hydrogen bonding between the polymer chains and surrounding water molecules. However, as the temperature surpasses the LCST, PNIPAM undergoes a coil-to-globule transition, expelling water from the polymer matrix and collapsing into a more compact structure, thereby reducing the overall nanoparticle size.

The observed *pH* and temperature-dependent behavior of the nanocomposite is rooted in the interplay between polymer protonation states of PIA and the thermoresponsive nature of PNIPAM, as represented in the heat map (Figure 4b). At a highly acidic *pH* of 2, the carboxyl groups in PIA become fully protonated and hydrophobic, converting –COO^−^ groups into –COOH groups. This protonation reduces the net negative charge along the polymer chains, thereby diminishing electrostatic repulsion between them and resulting in a compact polymer structure. Consequently, when the temperature is raised to 45°C, the PNIPAM segments again undergo a coil-to-globule transition, leading to a pronounced contraction of the polymer shell—a 32.32% reduction in hydrodynamic size. The red zones in the heat map (indicative of maximum swelling, ~1.3 µm) are prominent around *pH* 10 at lower temperatures, where PIA is deprotonated (–COO^−^ form), generating electrostatic repulsion and polymer chain extension.

At *pH* 4, partial protonation preserves hydration and network integrity, resulting in minimal shrinkage (1.49% size reduction) due to reduced electrostatic interactions and suppressed polymer chain collapse, which indicates limited temperature sensitivity. In contrast, at *pH* 10, the carboxyl groups on the PIA chains are partially deprotonated, leading to a moderate density of negatively charged –COO^−^ groups. This partial ionization induces electrostatic repulsion among the polymer chains, causing them to adopt a more extended and swollen conformation. As a result, the nanoparticles display their largest hydrodynamic diameter at this *pH*, with prominent red zones in the heat map (Figure 4b) indicating maximum swelling (~1.3 µm) around *pH* 10 at lower temperatures. Notably, a 12.32% increase in nanoparticle size was observed, further confirming the balance between deprotonation and repulsion at this intermediate *pH*.

At *pH* 12, the carboxyl groups are fully deprotonated and exist as –COO^−^, which imparts a higher negative charge density along the PIA chains. While this would typically enhance repulsion and swelling, several counteracting effects arise at this high alkalinity. These include charge screening due to increased ionic strength, potential chain contraction or stiffening, and neutralization by counterions, all of which contribute to reduced chain expansion. Consequently, despite the higher charge, the particles exhibit a smaller hydrodynamic size than at *pH* 10. Additionally, under *pH* 12 conditions, the thermoresponsive collapse of the PNIPAM block upon heating is partially suppressed. This is because the strong electrostatic repulsion among fully ionized –COO^−^ groups counteracts the typical temperature-induced shrinkage, resulting in a comparatively smaller reduction in particle size—only 14.35%.

Furthermore, a temperature-dependent size reduction in hydrodynamic diameter was observed across all *pH* values below 10, confirming the strong influence of PNIPAM’s thermoresponsive behavior. The blue zones (Figure 4b) in the heat map notably appear under low-*pH* and high-temperature conditions, signifying the maximum particle contraction is reached due to the combined effects of protonation-induced compactness and PNIPAM collapse. However, at *pH* 10, the nanoparticles reached a critical stability point where size remained unaffected by further temperature changes, as illustrated by the red zones (Figure 4b). This behavior likely stems from an equilibrium between electrostatic repulsion and polymer solubility, effectively suppressing the typical LCST-driven collapse of PNIPAM. These findings underscore the dual-responsive nature of PIA-b-PNIPAM@Fe_3_O_4_ composite, which exhibits tunable swelling and deswelling properties depending on temperature and *pH*. Such adaptability makes them highly promising for applications in controlled drug delivery, stimuli-responsive nanocarriers, and environmental sensing, where the precise regulation of nanoparticle size and stability is essential.

Eyiler and Walters synthesized and characterized superparamagnetic Fe_3_O_4_ nanoparticles functionalized with a dual-responsive block copolymer, PIA-b-PNIPAM, via aqueous SI-ATRP [57]. The resulting PIA-b-PNIPAM@Fe_3_O_4_ nanocomposites demonstrated clear temperature-responsive behavior, with a size reduction of approximately 20 nm between 25 °C and 34 °C, and the phase transition was observed around 32 °C, corresponding to PNIPAM’s LCST. In comparison, this present study represents an improvement over the previous work [57], achieving a more pronounced a ~45% reduction in hydrodynamic size between 25 and 45 °C at a neutral *pH*. Moreover, unlike Walters’ report, the current system reveals strong *pH*-dependent swelling and shrinking, with maximum swelling (~1.3 µm) at *pH* 10 and substantial contraction (<900 nm) at *pH* 2 and 12. These variations can be attributed to differences in surface functionalization (via APTES), and also to polymer shell composition, modulating hydrophilicity, ionic interactions, and the thermal behavior of nanocomposites. This enhanced dual-stimuli responsiveness indicates greater tunability compared to previous reports. While both studies used similar analytical approaches and confirmed PNIPAM’s reversible transition from a hydrophilic to a hydrophobic state upon heating, the observed differences are attributed to variations in grafting density, particle size, the surface modification of SPIONPs via APTES, and the extent of magnetization reduction due to polymer coatings.

It is important to note that different stimuli-responsive systems have also been reported using PNIPAM with different polymers. Gao et al. [72] developed poly(NIPAM-co-N-[3-(dimethylamino)-propyl] methacrylamide (DMAPMA))/clay hydrogel bilayers, dual-responsive soft actuators that respond synergistically to *pH* and temperature. In their system, PNIPAM imparts thermoresponsive behavior (LCST ~32 °C), while DMAPMA introduces *pH* sensitivity via the protonation/deprotonation of its tertiary amine groups (*pK_a_* ~9.8). By varying the NIPAM/DMAPMA ratio across bilayers, asymmetric swelling was achieved, enabling reversible curling/uncurling motions. These hydrogel actuators were successfully used as soft manipulators and artificial muscles, capable of grasping objects or lifting weights when exposed to external stimuli. However, while Gao et al.’s hydrogel bilayers function as macroscale actuators, PIA-b-PNIPAM@Fe_3_O_4_ nanocomposites can operate in the nanoscale to low-micron regime (~500 nm–1.3 µm), making it more suitable for biomedical applications, particularly intra-articular drug delivery. Additionally, the incorporation of a magnetic Fe_3_O_4_ core introduces magnetic responsiveness, adding potential for MRI tracking, targeted delivery, and stimulus-enhanced retention. Deloney et al. [73] developed hollow, degradable poly(N-isopropylacrylamide) nanoparticles (200–1000 nm) featuring a core–shell design for the intra-articular delivery of MK2-inhibiting anti-inflammatory peptides. These carriers leveraged PNIPAM’s LCST behavior to regulate drug loading and release—swelling below the LCST to incorporate the drug and collapsing at body temperature to trigger release. In comparison, PIA-b-PNIPAM@Fe_3_O_4_ nanocomposites integrate *pH* responsiveness with similar hydrodynamic dimensions, making them highly suitable for intra-articular osteoarthritis applications. Collectively, these findings highlight the promise of PIA-b-PNIPAM@Fe_3_O_4_ nanocomposites for dual-stimuli-responsive drug delivery and biomedical applications.

## 4. Conclusions

A dual-responsive block copolymer comprising poly(itaconic acid) (PIA) and poly(N-isopropylacrylamide) (PNIPAM) was successfully grafted onto the surface of the APTES-functionalized Fe_3_O_4_ nanoparticles with an average diameter of ~208.54 nm using a hydrothermal method, ensuring uniform size distribution and surface functionalization. The structural and physicochemical properties of the nanoparticles were comprehensively characterized using transmission electron microscopy (TEM), Fourier transform infrared spectroscopy (FTIR), and dynamic light scattering (DLS). FTIR verified successful silanization of the nanoparticle surface by detecting characteristic Si–O–Fe and –NH_2_ functional groups, indicative of effective APTES (3-aminopropyltriethoxysilane) coating. The polymer grafting was also confirmed through FTIR spectra, showing characteristic vibrational peaks of both PIA (carboxylic groups) and PNIPAM (amide and isopropyl groups). The DLS results revealed that the polymer-grafted magnetic nanoparticles exhibited excellent dispersion and stability in aqueous solutions, which was attributed to the hydrophilic nature of the grafted polymer chains and the electrostatic repulsion provided by carboxylic groups. Temperature responsiveness was assessed by monitoring changes in hydrodynamic diameter across varying temperatures at constant *pH*. Clear thermoresponsive behavior was observed at different *pH* values: increasing temperature led to a reduction in particle size due to the collapse of PNIPAM chains above their lower critical solution temperature (LCST), resulting in a more compact nanocomposite. However, under alkaline conditions (*pH* ≥ 10), the effective diameter remained relatively stable even with temperature elevation, likely due to electrostatic repulsion among deprotonated carboxylate groups in the PIA segments, which counteracted the thermally induced collapse of PNIPAM. These dual-responsive polymers, capable of responding to changes in both temperature and *pH*, hold significant promise for applications in smart drug delivery and various biomedical fields.

## Data Availability

The data presented in this study are available on request from the corresponding author.
